# Seronegative Antiphospholipid Syndrome: A Challenging Case Report

**DOI:** 10.1002/ccr3.9585

**Published:** 2024-11-17

**Authors:** Eihab A. Subahi, Soha Aboukhalaf, Shahd Mohammedain, Sagda Sayed, Elrazi A. Ali, Mohamed Subahi, Ijaz Kamal

**Affiliations:** ^1^ Department of General Internal Medicine Hamad Medical Corporation Doha Qatar; ^2^ Department of Radiology Hamad Medical Corporation Doha Qatar; ^3^ Department of Psychiatry Hamad Medical Corporation Doha Qatar; ^4^ Internal Medicine One Brooklyn Health/Interfaith Medical Center Brooklyn New York USA; ^5^ Department of Internal Medicine Our Lady of Lourdes Hospital Drogheda Ireland

**Keywords:** antiphospholipid syndrome, hepatosplenomegaly, lymphadenopathy, pyrexia of unknown origin, stroke

## Abstract

Seronegative antiphospholipid syndrome (SN‐APS) is uncommon and challenging condition, which should be included in the differential diagnosis of stroke in young, since it can result in arterial thrombosis.SN‐APS is typically diagnosed by exclusion; however, it is crucial to recognize it in order to choose the best antithrombotic treatment to lower the recurrence rate.

## Graphical abstract

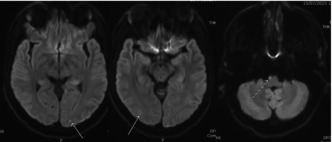

## 1 Introduction

**FIGURE 1:**
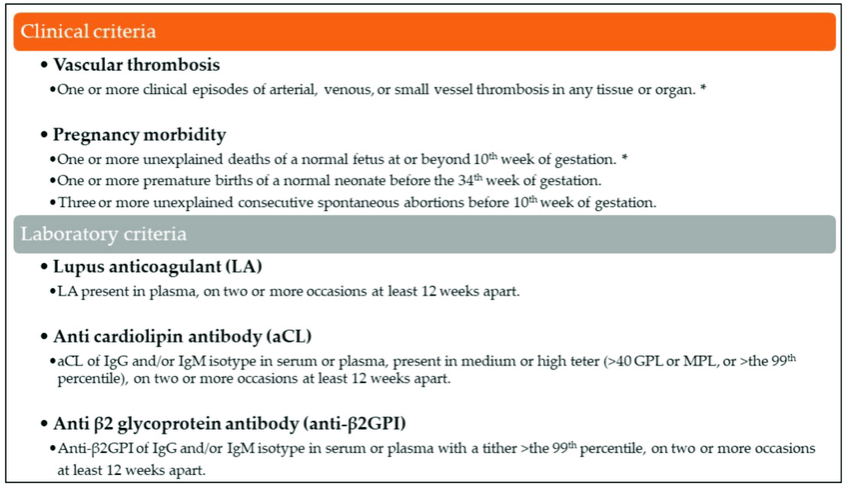
APS diagnosis criteria, updated at the Eleventh International Congress on Antiphospholipid Antibodies in Sydney in 2006. Antiphospholipid antibody syndrome (APS) is present if at least one of the clinical criteria and one of the laboratory criteria are met. Seronegative APS can be considered when a patient displays clinical features of APS with negative lab results. The clinical criteria that correspond the subject is marked (*).

Antiphospholipid syndrome (APS) is characterized by diffuse arterial and/or venous thrombosis, recurrent pregnancy loss, and persistently positive antiphospholipid antibodies (aPLs) [1]. The 2006 revised Sapporo criteria, which demands the presence of at least one clinical manifestation and one positive laboratory criterion, are the most recent categorization criteria for diagnosing APS [1] (Figure 1). After the Sapporo criteria were applied, disagreement developed since they excluded a category of APS patients known as seronegative APS (SN‐APS) in favor of identifying a more homogeneous group [1]. The primary definition of SN‐APS turned into, given in 2003 by Hughes and Khamashta, who described patients with medical manifestations incredibly suggestive of APS in absence of the laboratory standards inclusive of lupus anticoagulant (LAC), anti‐cardiolipin (aCL), and beta 2 glycoprotein (β2GPI) antibodies [1, 2]. Seronegative APS is often a diagnosis of exclusion and need to be suspected in patients with a scientific history suggestive of APS, along with people with recurrent arterial and venous thrombosis, recurrent miscarriage, or unexplained thrombocytopenia, with continual negativity of aPL tested on at least two occasions, and while other causes of thrombosis are excluded, consisting of genetic thrombophilia (element V and II mutations), active cancer, trauma, major surgery, or prolonged bed rest [2]. That is specifically glaring in younger patients without stablished cardiovascular risk factors (i.e., obesity, diabetes, hypertension, dyslipidemia). Most significantly other causes of coagulopathy should be excluded first, which include protein C, protein S, and antithrombin deficiency. In addition, the patient's medical history must be cautiously investigated to exclude preceding positivity to aPL [2]. In APS patients with previous arterial or venous thromboembolism, use of unfractionated or low molecular weight heparins (LMWH) is recommended in the acute phase, followed by long‐term treatment with warfarin, with an international normalized ratio (INR) range between 2 and 3 [2]. Seronegative APS is difficult and challenging diagnosis. We present here our personal experience with a patient who had history of multiple ischemic stroke and pyrexia of unknown origin (PUO) found to have generalized lymphadenopathy and hepatosplenomegaly but serological results for APS Abs were negative. This patient had a strong clinical suspicion of APS. Since SN‐APS is an extremely uncommon syndrome that is frequently overlooked in clinical settings, our capacity to diagnose APS may be enhanced by recently developed antiphospholipid (aPL) techniques. Nevertheless, aPL tests are not widely available in standard laboratory procedures. Actually, only a handful of labs regularly test for even aβ2GPI, and even fewer research labs screen for other aPL like anti‐prothrombin or anti‐annexin antibodies.

## 2 Case History/Examination

We report a 35‐year‐old Nepalese male who arrived at the emergency department with complaints of left‐sided vision loss and bilateral lower limb paralysis. He had no significant past medical or surgical history.

According to the patient, 2 days before to admission, he experienced a sudden painless loss of vision in his left eye, characterized as a total ‘black’ field with no pictures. While in the hospital, the patient experienced abrupt onset left lower limb weakness without concomitant sensory loss, which was followed by right sided weakness. Furthermore, the patient claimed being unable to void his urine bladder. The patient also acquired a fever in the hospital, with a maximum temperature of 39.3°C. He had no prior history of weakness or numbness in the limbs or loss of vision or seizures or syncopal episodes. He denied any chest pain, palpitations, weight loss, night sweats, joint pain, swelling, skin rashes, or oral ulcers.

On general examination, the patient was clinically and vitally stable, with the exception of a high‐grade fever. He appeared awake and well‐oriented. He was well‐nourished and not in any serious trouble. He was discovered to have bilaterally enlarged axillary and right posterior cervical lymph nodes. The examination revealed no jaundice, pallor, ness rash. The cardiovascular assessment was unremarkable. Abdominal examination revealed a painful suprapubic lump that was consistent with a dilated urinary bladder and was confirmed by ultrasonography. Foley's catheter was placed to remove 1.4 L of urine. An examination of the neurologic system revealed an aware and oriented individual with a Glasgow coma scale (GCS) of 15. The aberrant confrontation test revealed decreased visual acuity in the left eye. The cranial nerves were otherwise intact. His left upper arm had a power of 3/5, while both lower limbs had a power of 0. Neurological evaluation revealed no abnormalities in the right upper limb. The tone increased with the absence of reflexes in both lower limbs, but feelings and proprioception remained intact. No cerebellar symptoms were discovered during the evaluation.

## 3 Methods (Differential Diagnosis, Investigations, and Treatment)

An urgent computed tomography of head (CT head), CT perfusion, and CT angiogram were done immediately after presentation to the emergency department which showed no evidence of ischemic insult. Ophthalmological examination revealed left central retinal artery occlusion. The patient was admitted under medical team with stroke team consultation for further work up to ascertain the etiology of neurological deficit. This work up sought to rule out any vascular, infectious, malignant, vasculitis, or autoimmune causes.

Initial lab investigations upon admission revealed bicytopenia (Table 1). The patient had anemia with hemoglobin of 8.6 g/dL with mixed picture of iron deficiency and anemia of chronic disease (with normal B12 and folate values), in addition to leukopenia with white blood cells (WBC) of 3.2 × 10^3^/μL. The patient had normal kidney function tests, with only a reduced serum albumin value of 25 g/L on the background of normal liver function parameters (Table 2).

**TABLE 1:** Hematology lab results.

Lab	Value	Reference range
WBC	3.2 × 10^3^/μL	4–10 × 10^3^/μL
Hemoglobin	8.6 g/dL	13–17 g/dL
Platelets	501 × 10^3^/μL	150–410 × 10^3^/μL
MCV	60.2 fL	80–100 fL
Retic %	1.8%	0.5%–2.5%
Hgb A	93.4%	95.8%–98%
Hgb A2	5.7%	2.0%–3.3%
Iron	4 μmol/L	6–35 μmol/L
TIBC	45 μmol/L	45–80 μmol/L
Transferrin	1.8 g/L	2.0–3.6 g/L
Fe% saturation	9%	15%–45%

**TABLE 2:** Chemistry lab results.

Lab	Value	Reference range
Urea	4.8 mmol/L	2.5–7.8 mmol/L
Creatinine	72 μmol/L	62–106 umol/L
Sodium	131 mmol/L	133–146 mmol/L
Potassium	4.8 mmol/L	3.5–5.3 mmol/L
CRP	26.2 mg/L	0–5 mg/L
Procalcitonin	0.35 ng/mL	< 2.0 ng/mL

**FIGURE 2:**
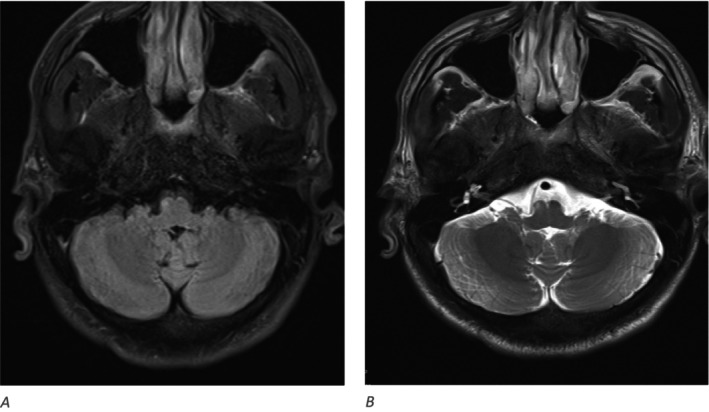
Hyper‐intense foci seen in right medullary pyramid in the FLARE image (A) with the corresponding T2 weighted image (B).

**FIGURE 3:**
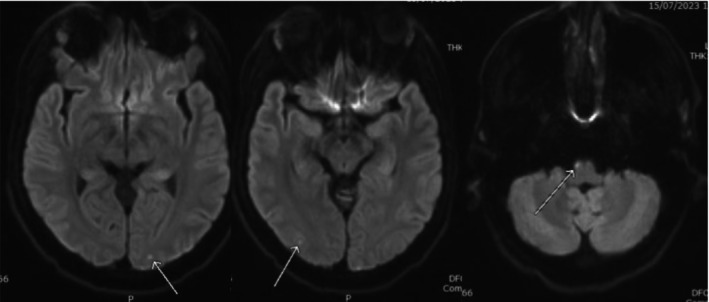
DWI images showing multiple tiny foci of restricted diffusion in the right medullary pyramid as well as bilateral occipital cortex suggestive of acute infarcts.

**FIGURE 4:**
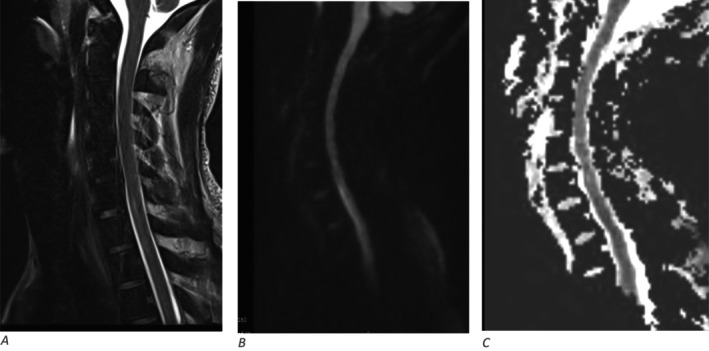
Linear area of T2 hyper‐intensity (A) with suspected diffusion restriction in DWI (B) and hypo‐intensity in ADC image (C) is seen at the anterior spinal cord at C6–C7 level suggestive of infarct.

**FIGURE 5:**
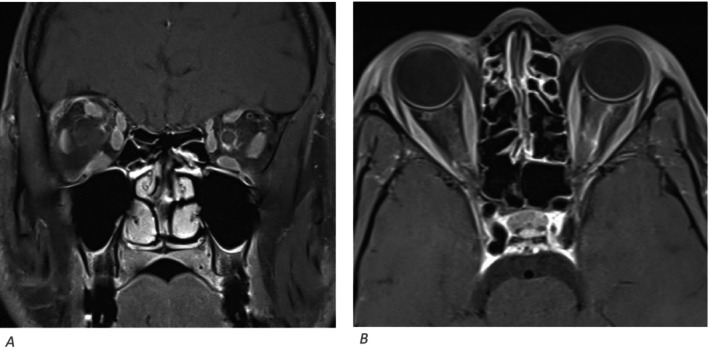
Thick concentric peri‐optic nerve enhancement is seen in T1 post contrast coronal (A) and axial cuts (B) suggestive of optic peri‐neuritis.

Magnetic resonance image (MRI) of the brain and whole spine was done as part of the work up and showed multiple recent infarcts in the right medullary pyramid (Figures 2 and 3), bilateral occipital cortex (Figure 3) as well as spinal cord at C6–C7 level (Figure 4) and left optic peri‐neuritis (Figure 5). Transthoracic echocardiography and extended Holter for 48 h were done to complete full stroke work up and yielded no significant findings.

Lumbar puncture was performed, and cerebrospinal fluid (CSF) analysis showed lymphocytic leukocytosis (total nucleated cells 9/μL, 95% lymphocytes) with normal protein (0.28 g/L) and normal glucose (3.34 mmol/L). Infectious work up, including bacterial, viral, fungal, was negative including cultures and viral polymerase chain reaction (PCR) (Table 3).

**TABLE 3:** Lumbar puncture lab results.

Lab	Value	Reference range
CSF WBC	9 (95% lymphocytic)	0–5 cells
CSF RBC	27	—
CSF glucose	3.34 mmol/L	2.22–3.89 mmol/L
CSF protein	0.28 g/L	0.15–0.45 g/L
CSF LDH	14.0 U/L	—
CSF IgG	80 mg/L	0–34 mg/L
Oligoclonal bands	Negative	—
Culture	Negative	—
CSF cryptococcal antigen	Negative	—
CSF TB PCR	Negative	—
CSF virology	Negative	—

During the hospital course, patient continued to spike fever despite negative septic work up including blood culture, urine, and CSF culture.

**FIGURE 6:**
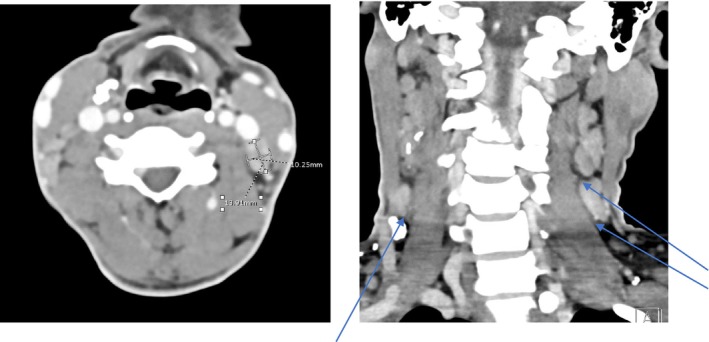
CT neck showing multilevel bilateral enlarged cervical lymph node shown in axial and coronal views.

In view of persistent fever with multiple CNS infarcts and no clear cause, pan CT was performed to rule out occult infection or malignancy as a possibly etiology. The CT of the neck, thorax, and abdomen revealed enlarged lymph nodes on both side of the diaphragm, in both groins, with hepatosplenomegaly and some lung lesions with differential diagnosis of malignancy, chronic infectious or granulomatous disease. Multiple calcific lymph nodes were also seen throughout chest and abdomen (Figure 6).

Full autoimmune work up revealed persistent bi‐cytopenia (anemia with lymphopenia), positive anti‐nuclear antibodies (ANA) (1:640), positive anti‐double strand DNA antibody (dsDNA Ab) (139.00 IU/mL), positive ribonucleoprotein (RNP) (29.0 U/mL), positive anti–Sjögren's syndrome‐related antigen A (SSA/RO) antibody (18.0 U/mL), positive Rib Ab (64.0 U/mL), with mildly elevated erythrocyte sedimentation rate (ESR) (29 mm/h), and C‐reactive protein (CRP) (55.5 mg/L). Complement levels revealed reduced C3 (0.47 g/L) with normal C4 (0.11 g/L). Coagulation profile showed prolonged PT (14.5 s), normal INR (1.2), and APTT (35.2 s) (Table 4). aPL serology was negative including anticardiolipin antibodies (aCL), anti‐beta 2 glycoprotein‐I (aβ2GPI), and lupus anticoagulant (LAC) (Table 5).

**TABLE 4:** Coagulation profile.

Lab	Value	Reference range
Prothrombin time	13.7 s	9.4–12.5 s
INR	1.2	< 1.5
APTT	40.3 s	25.1–36.5 s
D‐Dimer	1.90 mg/L	0–0. 49 mg/L
Fibrinogen	3.60 g/L	2.0–4.1 g/L
Factor V Leiden mutation	Not detected	—

**TABLE 5:** Autoimmune work up.

Lab	Value	Reference range
ANA	Positive (1:640)	—
Anti‐ds‐DNA	Positive (139)	—
Lupus screen	Negative	—
Anti‐cardiolipin IgM	Negative	—
Anti‐cardiolipin IgG	Negative	—
Anti B2 glycprotein	Negative	—
Anti RNP	Positive (29 U/mL)	—
Anti Ro	Positive (18 U/mL)	—
Anti La	Negative	—
Anti Jo‐1	Negative	—
Anti Scl‐70	Negative	—
Anti SmD	Equivocal (9.6 U/mL)	
ANCA	Negative	—
C3	0.43 g/L	0.9–1.8 g/L
C4	0.08 g/L	0.10–0.4 g/L
Serum IgG	31.29 g/L	6.1–16.6 g/L
ESR	29 mm/h	2–28 mm/h

Urine analysis showed proteinuria with 24‐h protein of 0.61 g/24 h and urine protein ratio of 129.47 mg/mmol.

Further thrombophilia work up including antiphospholipid antibodies, protein C and S as well as anti‐thrombin level were all unremarkable. Similarly, hemoglobin electrophoresis for this patient showed normal hemoglobin pattern. Likewise, infectious work up including tuberculosis, brucellosis and syphilis serologies were all negative (Table 6).

**TABLE 6:** Infectious diseases work up.

Lab	Value	Reference range
HIV Ab/Ag	Non‐reactive	—
Brucella IgG/IgM	Negative	—
Treponema Ab	Non‐reactive	—
Hep B surface antigen	Non‐reactive	—
Hepatitis C Ab	Non‐reactive	—
Quantiferon	Negative	—

The Rheumatology team was also involved with consideration of systemic lupus erythematous (SLE) as a diagnosis although clinical features were atypical for SLE. A transesophageal echocardiogram was performed with no evidence of intracardiac thrombi or vegetations.

The presence of positive anti‐dsDNA, low complement level, lymphopenia, hypoalbuminemia, and proteinuria made SLE a very likely diagnosis, even with the absence of typical SLE symptoms. However, without positive aPL serology, SLE alone could not explain the patient's multiple infarcts.

The Hematology team was consulted as well and a lymph node biopsy was done to exclude any hematological malignancy as a possible underlying etiology, in view of the extensive lymphadenopathy seen on pan CT. Lymph node excisional biopsy showed reactive cellular changes with plasmacytosis and foci of Langerhans cells which can be seen in autoimmune disease but is not specific. The kidney biopsy, which was arranged in order to rule out lupus nephritis, was negative.

**FIGURE 7:**
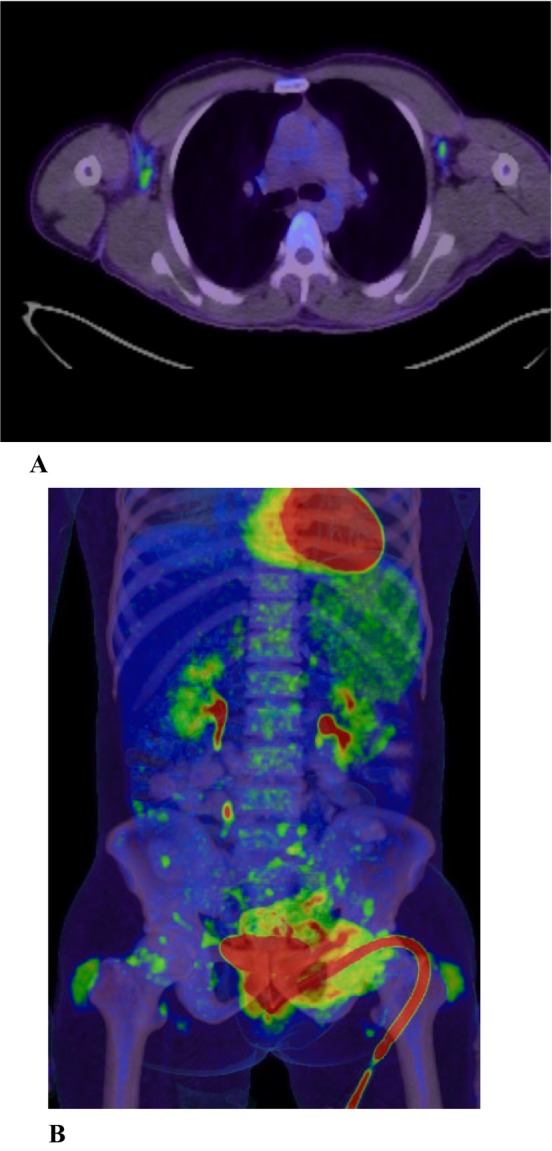
(A) PET CT showing slightly enlarged moderately hypermetabolic lymph nodes are seen in both axillary regions. (B) CT showing Hepatosplenomegaly. Hyperactive spleen and bone marrow. Mildly enlarged lymphadenopathy in the abdomen. Overall activity in these lymph nodes is of mild degree. The findings may indicate a low‐grade lymphoma with differential diagnosis of systemic infection.

**FIGURE 8:**
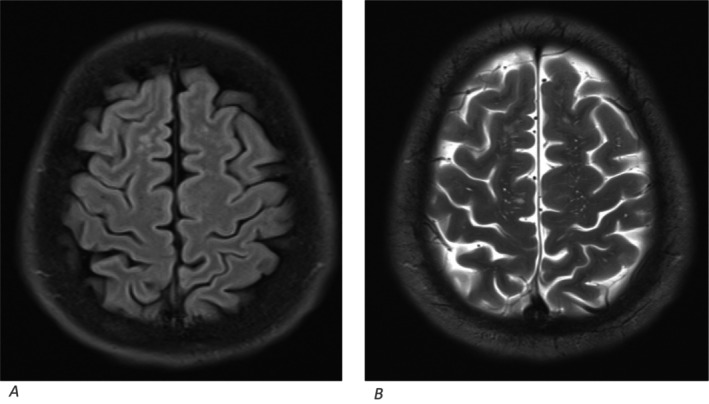
Multiple T2 bright spots are seen in the frontal parietal subcortical regions bilaterally in the FLARE image (A) with the corresponding T2 weighted image (B).

**FIGURE 9:**
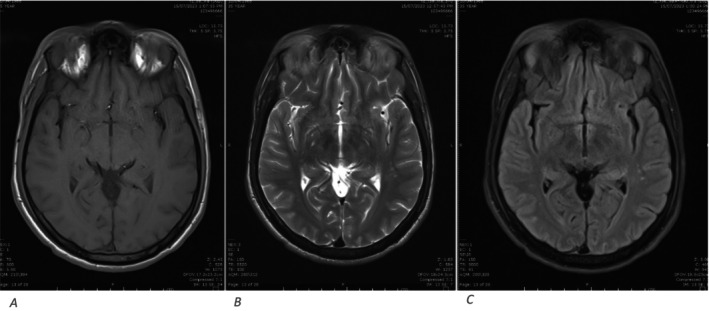
Hyperintense foci are seen in the occipitotemporal subcortical region in the FLARE image (C) with the corresponding T2 weighted image (B). while it appears isointense in T1 weighted image (A).

Further work up for vasculitis yielded negative antineutrophil cytoplasmic antibody (c‐ANCA) and perinuclear (p‐ANCA) values. Positron emission tomography (PET) scan was done which did not show any evidence of vasculitis (Figure 7). Additionally, the MRI images were reviewed with Neuro‐radiologist for any features of vasculitis, which was negative (Figures 8 and 9).

## 4 Conclusion and Results (Outcome and Follow‐Up)

The patient was thus initiated on hydroxychloroquine to manage his possible SLE. In the meanwhile, clinically, the patient continued to experience lower limb weakness and urinary incontinence, with no interval improvement despite ongoing physical and occupational therapy. Likewise, patient visual symptoms did not improve.

He continued to have periodic spikes of fever, without associated increase in inflammatory markers and no focus of infection, with negative multiple sets of blood and urine cultures. The fever was subsequently attributed to autoimmune process, and patient was started on steroids in addition to the hydroxychloroquine.

After ruling out malignancy and infection, the final working diagnosis for this patient was seronegative aPL syndrome in the context of multiple CNS infarcts with SLE‐positive serology, and he was started on warfarin.

Throughout his hospital course, our patient underwent extensive physical and occupational therapy, with gradual improvement in power to a strength of 3/5. Upon discharge, he was able to ambulate with walker for moderate distances and alternated between using a walker and wheelchair. However, no visual recovery was achieved. He was transferred to rehab unit with further follow‐up with the ophthalmology, rheumatology, and hematology teams.

In conclusion, seronegative APS is uncommon and challenging condition which, if missed, can lead to delay in diagnosis and management. Therefore, SN‐APS must be considered in young patients who has stroke after the exclusion of other causes of inherited and acquired thrombophilia conditions and particularly in patients who present with clinical manifestations of the syndrome, but persistently test negative for its accepted laboratory markers, namely aCL, LA, and aβ2GPI antibodies.

## 5 Discussion

APS is a systemic autoimmune disorder that can cause recurrent miscarriages during pregnancy and/or recurrent arterial and venous thrombosis when there is a consistently positive APL Abs [3]. Like classical APS, SN‐APS can have an accelerated progression resulting in multi‐organ failure, a life‐threatening medical condition known as catastrophic APS [1]. In the lack of laboratory criteria, such as LAC, aCL, and B2GP1 Abs, clinical signs strongly suggestive of APS were initially described as SN‐APS by Hughes and Khamashta in 2003 [1, 2, 3]. Antibodies against phosphatidylethanolamine, which has been linked to blocking the protein C pathway, phosphatidic acid, and phosphatidylserine—a phospholipid with a structure similar to cardiolipin but with serine added in place of the second glycerol group—as well as phosphatidylinositol, vimentin/cardiolipin complex, and annexin A2 and 5—are the most frequently studied Abs [3]. Additionally, up to 30 distinct non‐criteria autoantibodies that target glycoproteins, phospholipids, and coagulation factors have been found to be positive and may be associated with an increased risk of thrombosis in patients with APS [3]. Unfortunately, standardized assays to determine these Abs' levels are still lacking [3]. According to a 2020 study on SN‐APS diagnosis based on non‐criteria Abs, SN‐APS diagnoses should be established only when other potential causes of inherited and acquired thrombophilia disorders have been ruled out [3, 4]. The EULAR Guidelines propose treating SN‐APS in a manner comparable to treating seropositive APS [3, 5]. Our patient was diagnosed with systemic lupus erythematous (SLE) due to presence of positive anti‐dsDNA, low complement level, lymphopenia, hypoalbuminemia, and proteinuria, although clinical features were very atypical for SLE. However, without positive aPL serology, SLE alone could not sufficiently explain the patient's multiple infarcts. After ruling out malignancy, infection and other thrombophilia, the final working diagnosis for this patient was seronegative APS in the context of multiple CNS infarcts with SLE‐positive serology, and he was started on warfarin. The established risk factors of cardiovascular events and atherosclerosis are less linked to stroke in young adults. APS is one of the main risk factors of stroke in young adults. Previous research, however, only identified the thrombus formation pathway as the cause of stroke in individuals with APS; endothelial dysfunction brought on by autoantibodies is considered the “first hit,” with an inflammatory response following as the “second hit” [6]. Taking these aspects into account, we think that the patient's non‐criteria antibodies may have set off the various APS mechanisms and their intricate interactions, resulting in cerebral artery enormous thrombus formation [6]. Similar to this case, even though the technique for detecting non‐criteria antibodies is still in its early stages of development and was not available at our laboratory, SN‐APS should be regarded as a differential diagnosis in individuals who exhibit a typical course of the disease.

## Author Contributions

**Eihab A. Subahi:** investigation, methodology, resources, writing – original draft, writing – review and editing. **Soha Aboukhalaf:** investigation, resources, writing – original draft. **Shahd Mohammedain:** investigation, resources. **Sagda Sayed:** resources. **Elrazi A. Ali:** resources. **Mohamed Subahi:** resources. **Ijaz Kamal:** supervision, writing – review and editing.

## Ethics Statement

Written informed consent from the patient for the publication of his case.

## Conflicts of Interest

The authors declare no conflicts of interest.

## Data Availability

The data supporting this study's findings are available from the corresponding author upon reasonable request.
